# Subjective Stroke Impact and Depressive Symptoms: Indications for a Moderating Role of Health-Related Locus of Control

**DOI:** 10.3389/fpsyt.2019.00918

**Published:** 2019-12-19

**Authors:** Miriam Zirk, Vera Storm

**Affiliations:** ^1^ Department of Movement Science, Institute of Sport and Exercise Sciences, University of Münster, Münster, Germany; ^2^ Department of Exercise Psychology, Institute of Sport and Exercise Sciences, University of Münster, Münster, Germany

**Keywords:** stroke, rehabilitation, locus of control, depression, stroke impact

## Abstract

**Background:** Stroke is one of the most common causes of death and disability worldwide. Subsequently, depression occurs in about one third of the stroke survivors. Health-related locus of control might serve as a modifiable cognitive factor to aid the recovery process.

**Aims:** The present study aims to explore the role of health-related locus of control in the relation between subjective stroke impact and depressive symptoms after stroke.

**Methods:** In the present cross-sectional study, *N* = 44 stroke patients (*n* = 18 female and *n* = 24 male) were recruited at two rehabilitation centers in Germany. Mean age was *M* = 65.8 (SD = 11.52, Range = 48-88). Self-report instruments included the stroke impact scale, health-related locus of control questionnaire, and Beck depression inventory. Data analysis was conducted in R using correlation and regression methods.

**Results:** Subjective stroke impact and depressive symptoms were not directly associated (*r*_s_ = -.20, *p* = .216). However, health-related locus of control moderated the relationship between subjective stroke impact and depressive symptoms (*β =* -0.42, *p = .*015), revealing a stronger negative relationship when control of one`s health was attributed externally.

**Conclusion:** The results indicate that health-related locus of control plays a role in the relationship of subjective stroke impact and depressive symptoms. It is recommended to focus on control beliefs during the recovery process of stroke survivors. For developing targeted interventions, it is necessary to further investigate these associations while incorporating various health-related control belief concepts and replicate the present findings among larger samples.

## Introduction

Depression is one of the most frequent mental disorders following stroke and it affects approximately one third of stroke survivors during the first year after stroke and beyond (1, 2). Although post stroke depression has been recognized and investigated for several decades (3), the proportion of stroke survivors developing depression remains alarmingly high (2). Post stroke depression may affect patients' recovery process negatively and has been associated with poorer health outcomes and higher mortality rates (4, 5).

There is a reciprocal relationship between depression and physical function after stroke. On the one hand, more severe physical disability increases the risk for developing post stroke depression. On the other hand, depression has a negative effect on functional outcomes as depressive patients are less likely to participate in the rehabilitation process thereby not achieving best possible recovery (5). In particular, Gillen etal. (6) indicate that the use of rehabilitation services is less efficient regarding functional disability in patients with higher levels of depressive symptoms compared to those with lower symptom levels.

As outlined by Bonetti and Johnston (7), increased perceived control over one`s health will result in a higher frequency of behavior performance, and, consequently, better health outcomes. Control beliefs can be conceptualized as “locus of control” in the Social Learning Theory (8) or “perceived behavioral control” in the Theory of Planned Behavior (9). Health-related locus of control can be either attributed externally (believing that health is determined by factors in the surroundings such as fate or chance) or internally (believing that health is determined by oneself).

So far, only a few studies examined the relationship between perceptions of control and disability after stroke. Findings support the idea that an internal health-related locus of control over recovery is positively related to physical functioning after stroke, even when allowing for the current level of disability (10–12). Research on other patient populations with chronic diseases provides us with results indicating that there are associations between health-related locus of control and mental outcomes, such as depressive symptoms (13, 14). However, so far, there is only limited evidence that health-related locus of control might also be related to depression in stroke survivors (15). Literature does not report findings concerning a potential influence of health-related locus of control on the relationship of subjective stroke impact and depressive symptoms after stroke.

Given the fact, that health-related locus of control is a modifiable cognitive construct, its role in the perceived disability-depression relationship should be investigated as it might serve as an important factor when designing psychological interventions for stroke survivors. Therefore, in the present study, we test whether subjective stroke impact is positively related to depressive symptoms in stroke patients (hypothesis 1) and whether health-related locus of control moderates the relationship between subjective stroke impact and depressive symptoms (hypothesis 2) in stroke patients.

## Materials and Methods

### Study Design and Participants

The present pilot study has a cross-sectional study design. The study received ethical approval by the ethics committee of the University of Münster (Department of Sport Sciences and Psychology) on February 14, 2018, and has been preregistered within the open science framework (OSF) on https://osf.io/w7jex/.N=44 patients who had suffered from a stroke (ischemic or hemorrhagic) were recruited between April and July 2018 by two trained research assistants at two neurologic and orthopedic state-funded rehabilitation centers (inpatient and outpatient) in Germany. Participants were approached in the waiting area of the respective rehabilitation center. Stroke survivors meeting the following inclusion criteria were eligible to participate in the study: Being at the age of 18 or older, being able to communicate in German, self-reported occurrence of a stroke event, and being able to differentiate between the left and right side of the body. Exclusion criteria were: Inability to understand spoken language or communicate autonomously with the investigating person and blindness. After being informed about the study, eligible participants provided written consent.

### Measurements

This study employed a self-report questionnaire-based survey that consisted of three questionnaires, sociodemographic information, handedness, and passed time since stroke event. The questionnaires had been chosen based on their suitability to gather data for answering the research questions. Also, evidence-based quality characteristics for the application in German language were taken into account for the choice of questionnaires.

#### Subjective Stroke Impact

Subjective stroke impact was measured using the German short version of the Stroke Impact Scale (SIS-SF) by Petersen etal. (16). The German SIS is a reliable and valid instrument to assess physical deficits and health-related quality of life, which has been tested for the separate subscales (16). Reliability was found to be acceptable based on the current data of the present study (*Cronbach's alpha* = .74). By the SIS-SF the patient is requested to evaluate 8 items on how the stroke has impacted health and life from the own point of view, e.g., “In the past week how would you rate the strength of your leg that was most affected by your stroke?” Each item is rated on a 5-point Likert scale where the patient rates the difficulty for completing the item, for example with the answer 1 = “No strength at all” or 1 = “Extremely difficult” up to 5 = “A lot of strength” or 5 = “Not difficult at all.” For analysis the SIS-SF index was calculated by summing scores of the 8 items and then standardizing the score on a scale of 0 to 100 (17). Higher values indicate better self-reported health.

#### Health-Related Locus of Control

Health-related locus of control was investigated using the short version of the German questionnaire Fragebogen zur Erfassung gesundheitsbezogener Kontrollüberzeugungen (FEGK) by Ferring (18). Both subscales for internal and for external health-related locus of control consist of 5 items and show acceptable to good internal consistency based on the present study's data (internal: Cronbach's alpha = .74, external: Cronbach's alpha = .80). A statement from the internal subscale is for example “Good health depends on how looking after the own body,” a statement from the external subscale is for example “People who never fall ill are just lucky.” Each statement is judged on a 6-point Likert scale ranging from “very right” to “very wrong.” For both subscales the means were calculated. The higher the mean, the higher is the internal or external health-related locus of control, respectively (18).

#### Depressive Symptoms

The degree of depressive symptoms was obtained by the German Beck Depression Inventory (BDI-II) by Beck etal. (19). Internal consistency was demonstrated to be acceptable on the basis of the current data from this study (Cronbach's alpha = .75). Each of the 21 items has a set of at least four possible responses, ranging in intensity. For example, “I do not feel sad.” (0), “I feel sad” (1), “I am sad all the time and I can't snap out of it.” (2) or “I am so sad or unhappy that I can't stand it.” (3). According to the BDI-II manual, the cut-offs are 0 to 13 for minimal depression, 14 to 19 for mild depression, 20 to 28 for moderate depression and 29 to 63 for severe depression (19). For data analysis in the present study the sum score of the BDI-II was used. The BDI-II has proven valid among other clinical populations (20) including stroke (21, 22).

### Data Analysis

Data analysis was conducted in R (RStudio version 1.1.456) using descriptive statistics (e.g., Spearman correlations) and regression analysis. No corrections for multiple testing were applied, because the hypothesis was based on separate reasoning and has an explorative nature. Given the awareness that there is a possibility for false positive findings, the statistical significance level was set at *p* < .05.

## Results

### Descriptive Results

The study population consisted of *n* = 18 female and *n* = 24 male persons. The average age of the participants was *M* = 65.8 years (*SD* = 11.52) and *n* = 30 participants (71.4%) were married. The time passed after the stroke event when the participants were investigated lay between half a month and 19 years (in case of one patient who came every two years to the rehabilitation center for the treatment of his lasting severe physical impairments). Considering the strokes that occurred within the past year (*n* = 37) the average time since the stroke event was *M* = 2 months (*SD* = 1.43). *N* = 36 stroke patients had either an impaired arm or leg or both affected by the stroke, while *n* = 6 patients had no disability of their extremities. *N* = 25 stroke patients (60%) had no depressive symptoms at the time of questioning, *n* = 8 patients (19%) had minimal symptoms, *n* = 8 patients (19%) had mild symptoms, and *n* = 1 patient (2%) had moderate depressive symptoms. **Table 1** provides an overview of the main study variables.

**Table 1:** Mean (M), standard deviation (SD), Cronbach's alpha of the study variables, and participant number with missing data of a single questionnaire item.

	Number of items	*Min*	*Max*	*M*	*SD*	Cronbach's alpha	Data missing
SIS-SF	8	31,25	100	66.40	19.29	.74	6 (14,3%)
FEGK internal	5	1.8	5.6	4.74	0.82	.74	0
FEGK external	5	2.2	6.0	3.68	1.01	.80	0
BDI-II	21	0.0	25.0	7.98	5.68	.75	4 (9,5%)

SIS-SF, Stroke Impact Scale Short Form; FEGK, Fragebogen zur Erfassung gesundheitsbezogener Kontrollüberzeugungen; BDI-II, Beck Depression Inventory-II.

### Main Results

Against what we had hypothesized (hypothesis 1), the results of the Spearman correlation with a two-tailed significance test indicated no significant correlation between self-reported stroke impact and depressive symptoms (*r*_s_ = -.20, *p* = .216).

To explore whether health-related locus of control moderates the relationship between subjective stroke impact and depressive symptoms (hypothesis 2), a multiple linear regression (see **Table 2**) was conducted including an interaction term between health-related locus of control and subjective stroke impact. The model accounted for 25.5% of the variance. **Table 2** displays that external locus of control significantly predicted depressive symptoms (*β =* 1.20, *p = .*014). When the mean value of the external locus of control score increased by one, the sum score on the BDI-II increased on average by *b =* 8.21.

**Table 2:** Multiple regression for prediction of depressive symptoms following stroke with subjective stroke impact as independent variable and health-related locus of control as moderating variable.

Variables	*B*	*SE B*	*β*_standardized_	*t*	*p*
(Constant)	-13.32	19.18		-0.69	.492
subjective stroke impact	0.36	0.27	1.23	1.35	.186
eloc	8.21	3.16	1.20	2.60	.014*
iloc	-3.02	3.13	-0.52	-0.96	.342
subjective stroke impact x eloc	-0.12	0.05	-0.42	-2.57	.015*
subjective stroke impact x iloc	0.04	0.04	0.01	0.84	.406

eloc, external health-related locus of control; iloc, internal health-related locus of control *p < .05.

**Figure 1:**
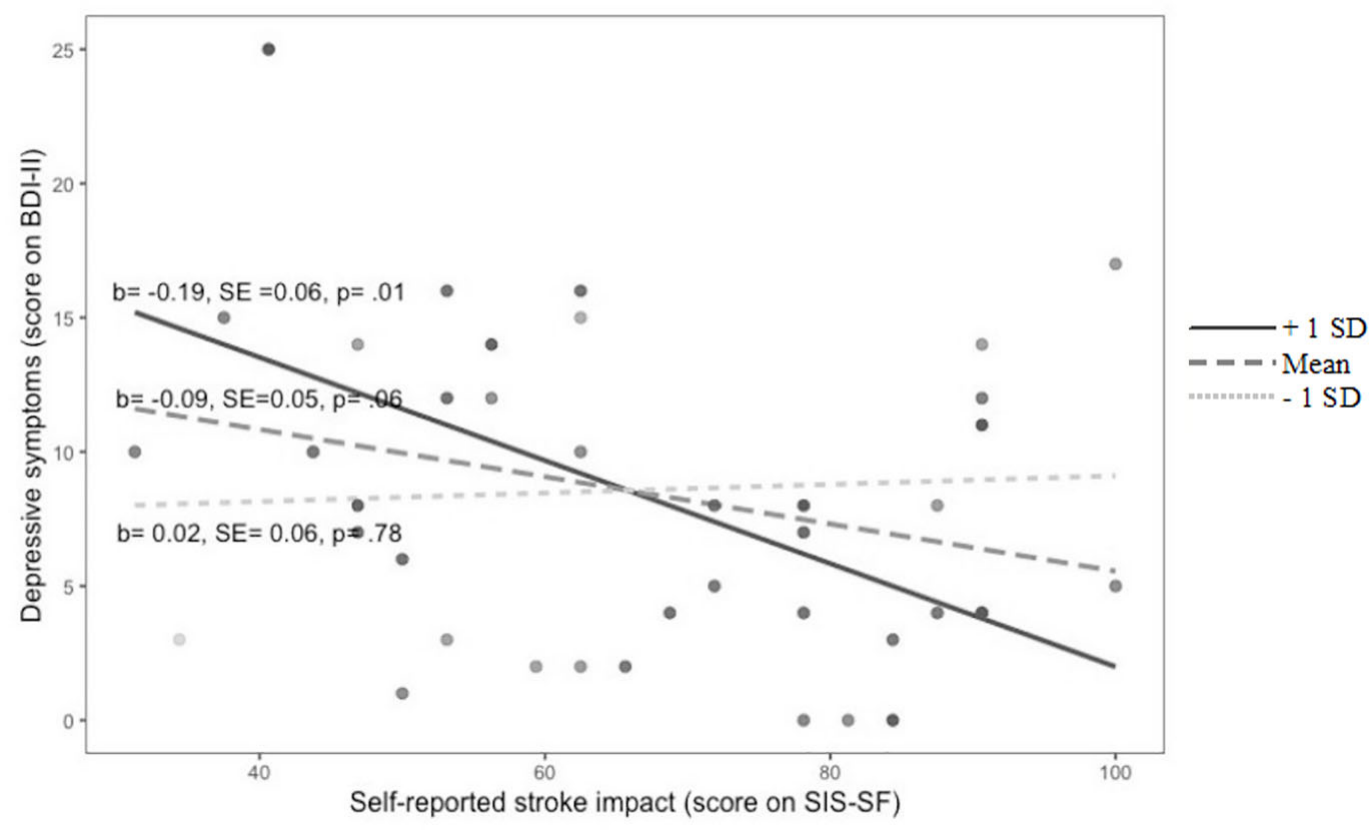
Moderation plot for the interaction of self-reported stroke impact and external health locus of control to predict depressive symptoms after stroke External health locus of control (score on FEGK): + 1 SD, Mean, - 1 SD.

In addition, the interaction between health-related locus of control and subjective stroke impact significantly predicted depressive symptoms (*β =* -0.42, *p = .*015). This indicates that external health-related locus of control significantly moderated the relationship between subjective stroke impact and depressive symptoms after stroke (see **Figure 1**).

The graph shows a significant difference of depressive symptoms amount in relation to subjective stroke impact for patients with a high external locus of control (+1*SD*, *p* = .01). There were no significant differences in terms of the relationship of subjective stroke impact and depressive symptoms for patients with both medium (*Mean*, *p* = .06) and low (-1*SD*, *p* = .78) external locus of control. When patients reported relatively low subjective stroke impact, those who had a high external locus of control had more depressive symptoms than they had when perceiving the stroke impact as more severe. However, post-hoc power analysis revealed low statistical power of these findings: Multiple R-squared of the regression model, power (1 - *β* error probability = 0.46).

## Discussion

There is a need to find treatable factors that address the mutual negative influence of disability and depression among stroke survivors. Therefore, the present exploratory study aimed to investigate the interaction of health-related locus of control with subjective stroke impact in relation to depressive symptoms.

No relationship between subjective stroke impact and depressive symptoms after stroke was found in the present sample. In addition, subjective stroke impact did not contribute to prediction of depressive symptoms when controlling for demographic variables. This is in contrast to existing evidence from the current literature. It has been consistently demonstrated that a reciprocal relationship between physical disability or functional recovery and depression following stroke does exist (1, 5).

A lack of association in the present sample might be due to limited sensitivity of the measuring devices utilized. Measurements for both self-reported stroke impact and depressive symptoms were based on self-report questionnaires. Although these questionnaires were shown to have good reliability and validity (16, 23), they are subjective assessments. Results may have been different if based, for example, on functional testing and examination by a psychologist like in other studies. It must be also kept in mind that further subjective and objective factors such as type and location of stroke play a role in the development of depressive symptoms after stroke (24). The prevalence of depression found in the present study was relatively low but still in a range that was reported in a previous systematic review (2).

In addition, results indicate that an external health-related locus of control appears to moderate the relation between subjective stroke impact and depressive symptoms following stroke. In those patients who attributed their health-related locus externally, stroke impact was less associated with depressive symptoms. Our findings also match the results from previous studies, in that lower perceptions of control, i.e. externality, were associated with more depressive symptoms (13–15). The interaction of external health-related locus of control and subjective stroke impact in the present sample, is indirectly in accord with previous findings that reported higher beliefs in personal control over recovery after stroke predict more functional recovery (7, 25). One possible explanation for patients having fewer depressive symptoms while perceiving severe stroke impact in combination with high external health-related locus of control in this sample might be a kind of protective mechanism that allows more acceptance of severe stroke impact due to lower perceptions of one's own control. As an internal attributional style for negative events is associated with depression (26), a high external health-related locus of control possibly leads to less negative feelings and reproaching of oneself. In fact, as no moderation effect of the internal health-related locus of control was found in this study, the meaning of health-related locus of control for the relationship of subjective stroke impact and depressive symptoms remains to be clarified.

The present study has a number of limitations. First, the investigated sample in this study was very small which reduces the power of the study. Therefore, the findings need to be interpreted with caution, and for future studies it is recommended to include a larger sample size to reach reliable results. Furthermore, the number of patients found to have depressive symptoms in the present study might be underestimated, because patients with cognitive impairments were excluded, while this is a risk factor for developing depression after stroke. In addition, it has to be taken into account that the timing of assessment after the stroke event may influence the number of patients classified as depressed (2). Finally, as this was a cross-sectional study, it could not provide any information of the impact of health-related locus of control on the relationship of self-reported disability and depression.

In summary, there is a small piece of evidence to support for a relevant role of health-related locus of control in the relationship of subjective stroke impact and depressive symptoms. There is a suggestion to take the treatment of health-related locus of control into account during the recovery process of stroke survivors. For developing targeted interventions, it is necessary to further investigate these associations in experimental designs with bigger samples while incorporating various health-related control belief concepts.

## Data Availability Statement

The raw data supporting the conclusions of this article will be made available by the authors, without undue reservation, to any qualified researcher.

## Ethics Statement

The studies involving human participants were reviewed and approved by Ethics committee of the University of Münster (Department of Sport Sciences and Psychology). The patients/participants provided their written informed consent to participate in this study.

## Author Contributions

MZ and VS made substantial contributions to the conception or design of the work. MZ was responsible for the analysis of data for the work. MZ drafted the work and, together with VS, revised it critically for important intellectual content. Both authors provide approval for publication of the content. Both authors agree to be accountable for all aspects of the work in ensuring that questions related to the accuracy or integrity of any part of the work are appropriately investigated and resolved.

## Conflict of Interest

The authors declare that the research was conducted in the absence of any commercial or financial relationships that could be construed as a potential conflict of interest.
